# Risk factors to identify patients who may not benefit from whole brain irradiation for brain metastases - a single institution analysis

**DOI:** 10.1186/s13014-019-1245-9

**Published:** 2019-03-11

**Authors:** Rebecca Buecker, Zhen-Yu Hong, Xiao-Mei Liu, Gert Jaenke, Ping Lu, Ulrich Schaefer

**Affiliations:** 1 Department of Radiotherapy, General Hospital of Lippe, GmbH Rintelner Straße 85 |, Lemgo, Lippe Germany; 2 Department of Radiotherapy, The First Affiliated Hospital of Xinxiang Medical University, Weihui, Henan China

**Keywords:** Whole brain irradiation, Brain metastasis, Median survival, Risk factor

## Abstract

**Background:**

Radiotherapy plays a major role in the management of brain metastases. This study aimed to identify the subset of patients with multiple brain metastases who may not benefit from whole brain irradiation (WBI) due to a short survival time regardless of treatment.

**Methods:**

We analyzed a total of 339 patient records with brain metastases treated with whole brain radiotherapy from January 2009 to January 2016. External beam radiotherapy techniques were used to deliver 33 Gy in 11 fractions (4 fractions per week) to the whole brain. Eight clinical factors with a potential influence on survival were investigated using the Kaplan-Meier method. All factors with a *P* < 0.05 in univariate analysis were entered into multivariate analysis using Cox regression.

**Results:**

In the present series of 339 patients, median survival time was 2.5 months (M; range, 0–61 months). Four risk factors Karnofsky Performance Score (KPS) < 70, age > 70, > 3 of metastases intracranial, uncontrolled primary tumor) were identified that were significant and negatively correlated with median survival time. Patients with no risk factors had a median survival of 4.7 M; one risk factor, 2.5 M; two risk factors, 2.3 M; and 3–4 risk factors, 0.4 M (*p* < 0.00001).

**Conclusions:**

Patients with identified risk factors might have a negatively impacted overall survival after WBI. Accordingly, patients who will not benefit from WBI can be easily predicted if they have 3–4 of these risk factors.

## Background

Brain metastases (BM) occur in 20–40% of all cancer patients and are more frequent than primary brain tumors [1, 2]. Whole-brain irradiation (WBI) alone is the most common treatment for these patients, resulting in a median survival of < 6 months in most series [3]. The aim of treatment in this situation is to palliate current neurological symptoms, prevent further symptoms, improve quality of life (QOL) and increase overall survival. WBI is not without acute toxicity as patients receiving WBI may experience acute side effects, such as headache, erythema, drowsiness, fatigue, nausea and dry desquamation of the scalp, as well as temporary alopecia; these symptoms may never resolve. The development of brain metastases signifies a poor prognosis and, if left untreated, median survival is around 1 month [4]. Thus, patients who die within 4 weeks of assessment (defined as ‘early death’) may not benefit from WBI; they may instead only experience a decline in their QOL. If identified at time of assessment, patients who are likely to experience an ‘early death’ may be better managed with steroid therapy and supportive care.

The purpose of this study was to evaluate the patient risk factors for ‘early death’ in a population with brain metastases treated with WBI during a 7-year period. By calculating the number of correlative risk factors, we aimed to identify those patients who were likely to experience ‘early death’ and, therefore, may not benefit from whole brain irradiation (WBI).

## Methods

This study was a retrospective analysis of 339 medical records of patients who received WBI alone without additional surgery or radiotherapy for brain metastases between January 2009 and January 2016. The Department of Radiation Oncology at General Hospital Lippe Germany maintains the electronic database of all patients that was used in this study. The diagnosis of brain metastases was confirmed by CT or MRI and other patient characteristics are summarized in Table 1. The univariate analyses of survival (from date of last WBI to occurrence of death) were performed with the Kaplan–Meier method and the log-rank test [5]. In this analysis, the following nine potential prognostic factors were investigated with respect to survival following WBI: age (30–49 years vs. 50–69 years vs. 70–90), gender, Karnofsky Performance Score (KPS < 70 vs. 70 ≤ KPS ≤ 80 vs. 80 ≤ KPS ≤100), type of primary tumor (breast cancer vs. NSCLC vs. SCLC vs. other tumors), time of occurrence, hospitalization, number of brain metastases (singular / solitary vs. ≤3 vs. > 3 or bulk). These potential prognostic factors were firstly analyzed with the Kaplan-Meier method. The differences between the Kaplan-Meier curves were determined with the log-rank test (univariate analysis). The prognostic factors found to be significant in the univariate analysis (*p* < 0.05) were then included in a multivariate analysis, performed with the Cox proportional hazards model. External beam radiotherapy techniques delivered 33 Gy in 11 fractions (four fractions per week) with combined beams of 6 and 15 MV photon beams from a linear accelerator to the whole brain. Patents did not receive any chemotherapy during WBI or 4 weeks before or after WBI. Those who had received previous brain radiotherapy, including prophylactic cranial irradiation and stereotactic radiation treatment, were excluded.

**Table 1:** Patients’ characteristics

Variable	Frequency	Median survival	Log Rank
Gender			N.S.
Female	146	2.9 M	
Male	193	2.1 M	
Age			*p* = 0.0002
30–49	37	7.0 M	
50–69	184	2.5 M	
70–90	118	1.5 M	
KPS			*p* = 0.0001
90–100%	94	5.2 M	
70–80%	138	2.5 M	
< 70%	107	1.2 M	
Time of occurrence			N.S.
Synchronous with primary	130	2.8 M	
After primary	209	2.4 M	
Hospitalization			*p* = 0.0001
Yes	126	0.9 M	
No	213	3.5 M	
Number of brain metastases			*p* = 0.031
Singular/solitary	65	3.7 M	
≤ 3	75	2.6 M	
> 3 or bulk	199	2.3 M	
Primary disease status			*p* = 0.0244
Controlled primary	64	3.8 M	
Uncontrolled primary	275	2.3 M	
Tumor site (3 most common and other)			N.S.
NSCLC	148	2.5 M	
SCLC	59	2.4 M	
Breast cancer	47	2.9 M	
Other	85		

Demographic data of the 339 patients with univariate analysis of potential risk factors for median survival. *N.S.* no significant difference

## Results

**Fig. 1:**
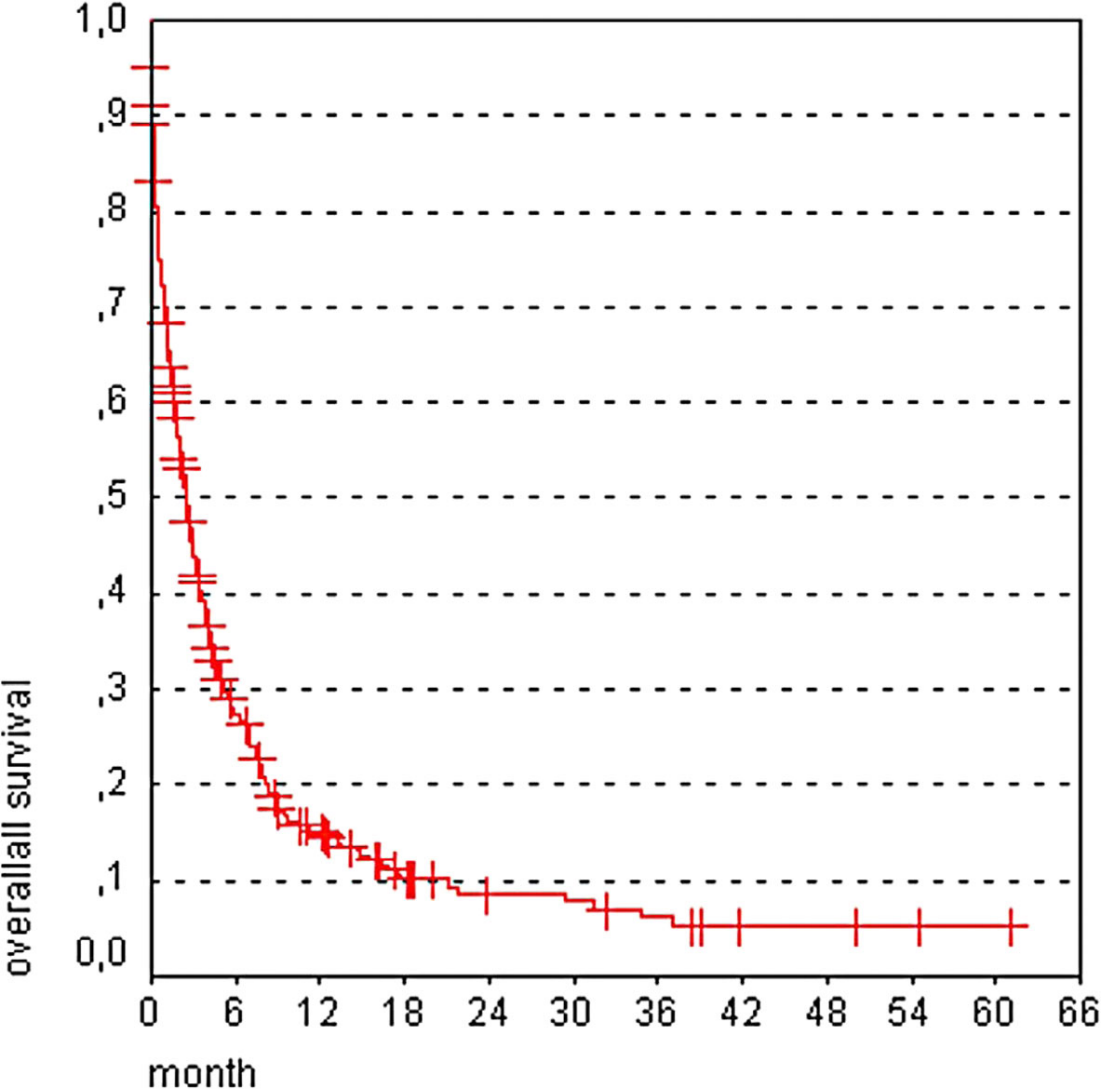
Overall survival after WBI. Of the 339 patients, the survival time was 0–61 months with a median survival time of 2.5 months

**Fig. 2:**
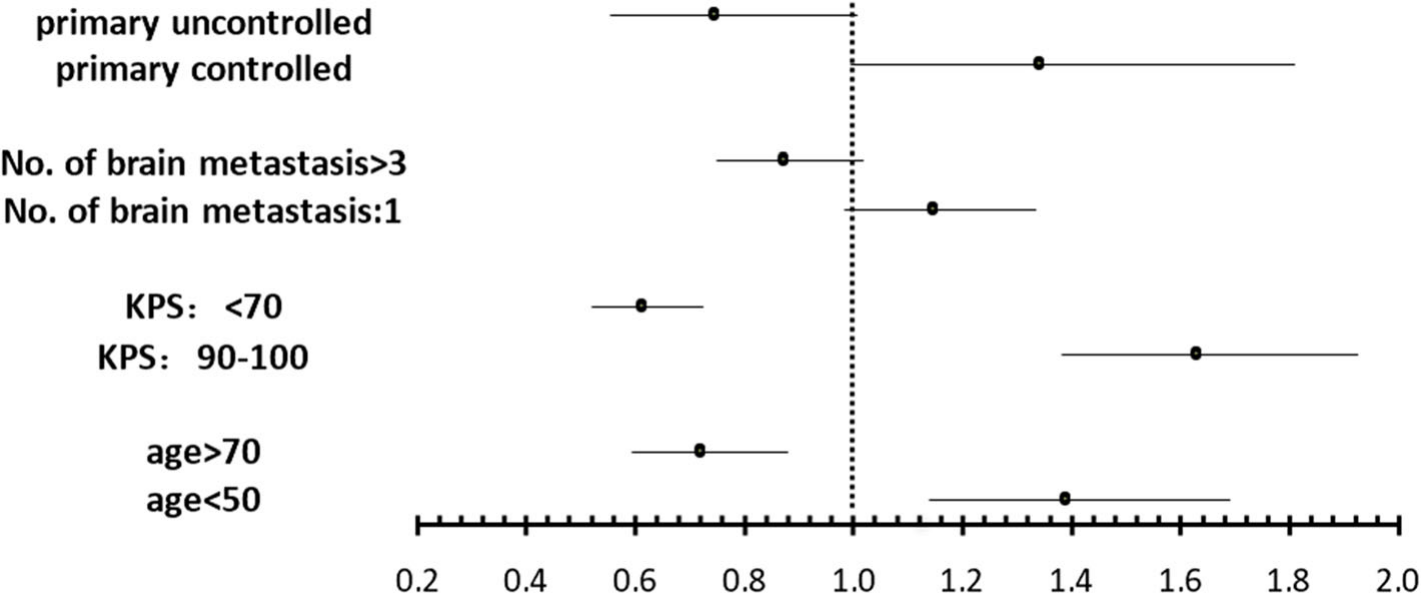
Odd ratio for overall survival with 95% confidence intervals. Odd ratio for overall survival: Risk factors with 95% confidence intervals

**Fig. 3:**
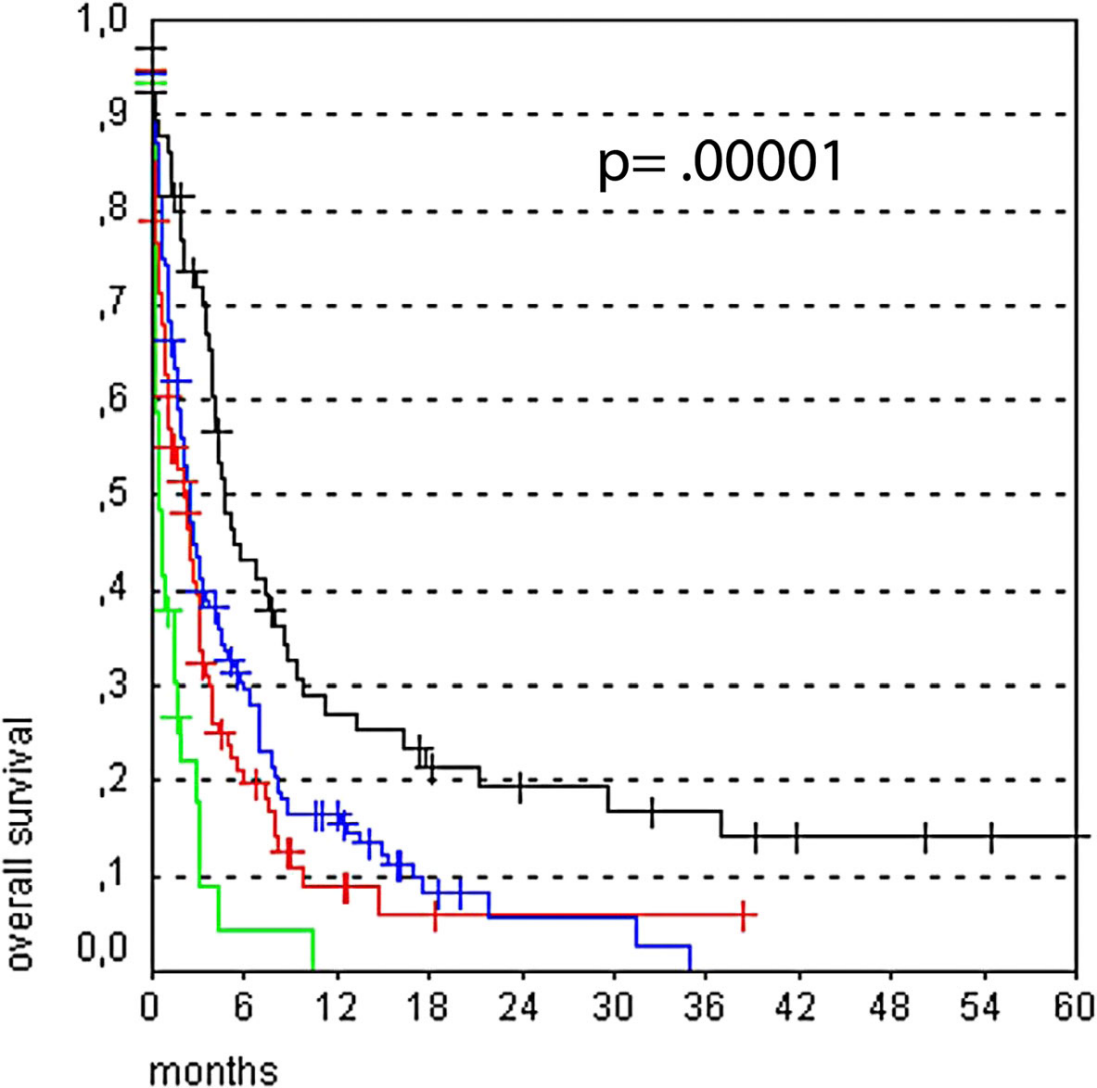
Overall survival according to the sum of risk factors from Fig. 2. Overall survival according to the sum of risk factors from Fig. 2: Black = 0, blue = 1, red = 2, green 3–4 risk factors

Patient survival time was 0–61 months (M) with a median survival time of 2.5 M (Fig. 1). Patient clinical characteristics and median survival are presented in Table 1. Our analysis revealed age > 70, KPS < 70 at baseline, uncontrolled extracranial tumor and number of brain metastases > 3 as significant prognostic factors for poor survival. After multivariate analysis (Cox regressions model), the following factors were still independently significant: age (*p* < 0.001), KPS at treatment start (*p* < 0.0001), number of brain metastases (*p* < 0.05), and extracranial tumor control (p < 0.05). Odds ratio for overall survival with a 95% confidence interval is presented in Fig. 2. Afterwards, these risk factors were summed and patients were grouped depending on the number of risk factors they had (0 risk factor, 1 risk factor, 2 risk factors, 3–4 risk factors) for further analysis. Median survival was strongly correlated with the sum of the independent risk factors listed in Fig. 2. Specifically, patients with no risk factors had a survival time of 4.7 M; 1 risk factor had 2.5 M; 2 risk factors, 2.3 M, and 3–4 risk factors, 0.4 M (*p* < 0.00001) (Fig. 3).

## Discussion

Most patients with brain metastasis from cancer have a poor prognosis [6, 7], but WBI can improve neurologic dysfunction and prolong life span of such patients [8]. At the other end of the spectrum, it is recognized that there is a subgroup of patients with brain metastases who have a relatively poor prognosis, and the routine use of WBI in the management of these patients is being increasingly questioned [9, 10]. Avoiding or minimizing the time commitment demanded by treatment and the QOL of patients and their families, particularly near the end of life, are issues especially for patients who will ultimately not benefit from the treatment. Inappropriate application of WBI also has resource implications for treating institutions and related health-care services.

In the current study, a considerable proportion of the patients recommended for WBI (18%) experienced an ‘early death’ and, probably, did not benefit WBI. This finding is similar to that of Bezjak et al. where 17% of patients died within 4 weeks of WBI [11]. Therefore, our findings justify the need to question the role of routine WBI and clearly support the need for an individualized approach to recommending WBI. An international randomized trial (QUARTZ trial) compared OSC (optimal supportive care) plus WBI and OSC alone may perhaps address this issue further [12]. According to their results, there was no evidence of a difference in overall survival, overall quality of life between the two groups. Improved survival with WBI was shown for younger patients, particularly those aged younger than 60 years. Other, non-significant, associations also suggested a potential survival benefit with WBI for patients with good performance status and a controlled primary NSCLC, although WBRT did not show a statistically significant benefit in these latter two groups.

Recognition of important risk factors will be valuable in making clinical decisions for individual brain metastasis patients. Various prognostic tools have been developed for this purpose. The most widely known tool is the Radiation Therapy Oncology Group (RTOG) recursive partitioning analysis (RPA), which classifies patients with brain metastases into three prognostic groups. This grouping system has been validated in subsequent RTOG and other trials [5, 13]. While it has been found to be useful in identifying the patients who have a relatively favorable prognosis (RPA class 1) and, therefore, may benefit from surgical metastatectomy or stereotactic radiation, that system has limitations in identifying the poor prognosis patients who have a high likelihood of ‘early death’. According to the RPA class groupings, patients with the worst prognosis (class III) had a median survival of 2–2.3 months and were classified into this class solely based on their performance status (KPS < 70) [5, 14]. The graded prognostic assessment (GPA) is a new prognostic index (PI) developed by the RTOG [15]. While GPA system has potential to improve on the RPA system by decreasing subjectivity, it again has limited utility in identifying the very poor prognosis patients. The GPA is the sum of scores (0, 0.5, and 1.0) for four risk factors which prognostic method seems similar to ours and it would explain why the survival curve showed in that study is similar to the present one. However, we found the worst subgroup of survival time identified in our study (0.4 month) is much shorter than GPA system (2.6 month). That might be partly caused by some of different risk factors in two studies (age > 70 vs. age > 60, status of primary control vs. extracranial metastases).

In order to identify the subset of lung cancer patients who may not benefit from WBI due to suffering ‘early death’ regardless of treatment, P Sundaresan et al. developed a prognostic index (PI) which introduced a prognostic factor weighting into its system [16]. It is difficult to make direct comparisons of our study to theirs as prognostic factors (except for ECOG) they analyzed were not significantly associated to OS (overal survival). In the present series of 339 patients, univariate and multivariate analysis of 8 potential prognostic factors for median survival showed that five of them (KPS, age, tumor control, number of metastases) were significantly different than medial survival. Furthermore, they focused on patients with lung cancer primary and assessed survival from time of WBI recommendation rather than from end of WBI. In our study, patients had different primary tumor sites and survival assessed from end of WBI. Although the PI proposed by Sundaresan et al. could be a valuable clinical decision tool, it is restricted to patients with lung cancer who had multiple brain metastases.

Barnholtz-Sloan et al. developed and internally validated an individualized prognostic nomogram for patients with brain metastasis [17]. Nieder et al. employed this nomogram to examine its ability to better predict short survival (cut-off 2 months) than previous models [18]. Despite the nomogram’s ability to stratify the patients into different prognostic groups, the survival curves of patients with intermediate point sum in the range of 90–139 points were largely superimposable in the Nieder et al. study. In contrast, its ability to predict poor survival was promising and comparable to the previously published models [19, 20]. Because nomogram is constructed by modeling the survival classification to determine the contribution of prognostic factors towards shortened survival, to a certain extent, it is complicated and tedious for clinicians to use it. Additional validation studies from different geographical regions as well as continuous monitoring of the models’ performance appear necessary to ensure their clinical applicability in the present era of rapidly changing treatment paradigms. More recently, except for BM-specific prognostic scores, Berghoff et al. provided a LabBM score based on standard hematologic and serum biochemical parameters that are useful for survival prediction of BM patients [21].

Among risk factors analysed in our study, hospitalization shows significant difference in survival by univariate analysis. However, it can be biased by the subjective decision of physicians and affected by the medical system. Considering it can’t be easily adopted by different medical systems, we ruled out the hospitalization as risk factor for further analysis.

Limitations of this study are lack of some risk factors such as extracranial metastases or recently revealed LabBM score et al. Despite of these limitations, the current study attempted to address the need for a simple, clinical decision method to objectively select patients in whom WBI may be avoided. At first, four risk factors (KPS < 70, age > 70, > 3 of metastases intracranial, uncontrolled primary tumor) were identified which had a statistically significant correlation with median survival. According to our further analysis, patients who will not benefit from WBI can be excluded from treatment if they have 3–4 risk factors.

## Conclusions

Our results demonstrate that KPS < 70, age > 70, > 3 intracranial metastases and uncontrolled primary tumor may be significant risk factors that impact on overall survival after WBI. Moreover, we found that patients who can benefit for WBI might be easily narrow down to the one who has < 3 of these risk factors.

## Abbreviations

BM: Brain Metastases
ECOG: Eastern Cooperative Oncology Group
KPS: Karnofsky Performance Score
M: months
N.S.: no significant difference
No.: number
PI: prognostic index
QOL: quality of life
RPA: recursive partitioning analysis
RTOG: Radiation Therapy Oncology Group
WBI: Whole-brain irradiation
